# Constant Daily Exercise to Keep the Doctor Away: A Study of Adherence to Physical Exercise Using a Gym in Individuals Older Than 55 Years

**DOI:** 10.1080/17482631.2020.1859174

**Published:** 2020-12-20

**Authors:** Ming-Hsin Chen, Ching-Len Yu, Su-Hsien Chang

**Affiliations:** a Department of Senior Citizen Services, National Tainan Junior College of Nursing, Tainan, Taiwan ROC; b Department of Environmental Engineering, Kun Shan University, Tainan, Taiwan ROC

**Keywords:** Physical exercise, qualitative study, middle-age people, older people, gyms

## Abstract

The purpose of this study was to examine the role of using a gym as a mechanism for adherence to regular physical exercise among individuals with aged 55 and over. This was a grounded theory research design. Data were collected from face-to-face interviews and observations among 15 people who attended the gym regularly and then analysed via content analysis. Three categories emerged from the present study regarding the adherence to regular physical exercise: (1) meaningful starting points, (2) reinforcement by motivators, and (3) adherence through positive changes. Results of this study can help health policymakers, managers of gyms/fitness centres, and gym instructors to design and implement programs

## Introduction

Adherence to regular physical exercise is greatly beneficial for one’s health. It is also a very challenging health behaviour. According to the World Health Organization (WHO, 2020), insufficient physical exercise is one of the 10 leading risks of mortality among people aged 18 and older. Individuals who participate in moderate-intensity exercise for at least 150 minutes per week have a 20%–30% decreased mortality risk in comparison with people who engage in insufficient physical exercise. Moreover, the benefits of regular physical exercise include a decreased risk of cardiovascular disease, improved blood pressure and lipid levels, and declined depressive mood status (WHO, 2020). Moreover, regular physical exercise significantly reduces body mass and results in weight loss (Innes et al., 2019). However, these benefits are still not appreciated by many people. In 2016, 23% of men and 32% of women aged 18 years and older worldwide did not engage in sufficient physical exercise (WHO, 2020). Geographically, the Western Pacific (19%) and African (22%) regions as well as low-income countries have the highest proportion of people engaging in insufficient physical exercise (WHO, 2020).

Several studies were conducted to identify barriers and facilitators of adherence to regular physical exercise. Some common barriers were observed, including symptoms of swollen, stiff, painful joints and swollen ankles and legs (Carpenter & Gilleland, 2016); outdoor temperature; poor neighbourhood conditions (Albrecht et al., 2020; Gothe & Kendall, 2016); time; peer pressure; and family responsibilities (Gothe & Kendall, 2016). In contrast, facilitators and motivators included one’s experiences of better health, well-being, and enjoyment and receiving social support (Alvarado et al., 2015; Gothe & Kendall, 2016). Hence, the experience of pressure, such as guilt around missing classes and fear of missing out, were strong motivators for consistent attendance at physical exercise programs (Alvarado et al., 2015).

Gyms have been a place dedicated to improving physical exercise in a controlled environment. Gyms typically offer a variety of indoor activities, amenities, and events, although users must pay to attend. As far as the authors are aware, an evaluation of the effect of gym use on adherence to regular physical exercise has not been established in particular for individuals aged 40 years and older. Thus, the purpose of this study was to examine the role of using the gym as a mechanism for adherence to regular physical exercise among individuals with aged 55 and over.

## Methods

A grounded theory research design was used to investigate the reasons some people attend a gym regularly and to determine their experiences, barriers, and facilitators towards gyms. The grounded theory was selected in that it can be used to explore the interrelationship between subjects’ actions and meaning in the perception of the subjects (Aldiabat & Navenec, 2011). The subjects included gym members aged 40 years and older. Data were collected from face-to-face interviews and observations and then analysed via content analysis.

### Setting and participants

A purposive sampling method was used. Subjects were recruited from three gyms located in the urban area of the southern Taiwan. Eligibility criteria included the following: (1) aged 40 years and older, (2) with a monthly or annual gym membership for 12 months or longer, (3) ability to speak Mandarin or Taiwanese, and (4) agreement to participate in a tape-recorded interview.

### Interviews

The face-to-face, in-depth, and individual interviews were conducted by the primary author (Ming-Hsin Chen) in a private, quite, and comfortable room with the goal of uncovering the views of each participant. Interviews began with an open-ended question: “What makes you adhere to regular physical exercise?” Each interview lasted from 30 to 60 minutes. All interviews were conducted in Mandarin or Taiwanese and tape-recorded. The interview data were transcribed verbatim prior to coding.

### Analysis

Translations of transcribed interviews were performed after data collection. According to Mariano (1995), data collection and data analysis and a “hand-in-hand” task, in that an initial data analysis could be used to guide the subsequent data collection. This reciprocal process continued until no new findings were found, that is, the data collection was complete. We adopted this “hand-in-hand” task approach in this study. In addition, this study used a three-step method of data analysis (Lincoln & Cuba, 1985). The three steps of data analysis included the following: disaggregating data into the smallest unit of information, labelling (coding) these units, and progressively sorting them into meaningful categories. This process was repeated for each interview; therefore, meaningful and accurate categories were reached.

The content analysis was used to analyse the data with original language. We analysed interview data into units, then summarized into categories. Next, they both discussed developing categories and coding scheme. After agreeing on the general categories and coding schema, the principle investigator (PI, Su-Hsien Chang) coded each interview. All disagreement was discussed until a consistency of coding employed. After most of data were analysed and the categories were clearly draw, identifying the relationship of uncover patterns coding was used to group categories into a smaller number of themes. The coding was performing with original language, then translated by two authors (the second author and PI).

The rigour of this study included three criteria: creditability, transferability, and dependability (Lincoln & Guba, 1985). To ensure credibility, the PI and study subjects read and discussed the meanings derived from the analysis. To establish transferability, the PI made observation notes during the interviews and recorded these. These observation notes were used to compare or clarify a particular subject’s transcript. To achieve dependability, all interviews were conducted by the PI and tape-recorded. Hence, the interview data were transcribed verbatim by a research assistant, and the accuracy of the transcription was re-examined by the PI.

### Ethical considerations

The study was approved by the Human Subjects Protection Programme at National Cheng Kung University, Tainan, Taiwan. To recruit the subjects, a sponsor or administrator of the gym first introduced the primary investigator (PI) to members when they attended the gym for physical exercise activities or classes. Next, the PI explained the purpose, rationale, and background of the study to the subjects. Gym members were encouraged to ask questions and participate in the study. If a member of the gym agreed to participate, she or he signed a consent form.

## Results

### Demographic information

Fifteen subjects participated in this study. Most were female (n = 13, 86.7%), and married (n = 14, 93.3%). The average number of children were 1.8 (minimum = 0; maximum = 3). The average age was 56.93 years (minimum = 68.83; maximum = 46.45), the average length of time of attending the gym was 15.24 years (minimum = 3.33; maximum = 28), and the average number of hours spent doing physical exercise weekly was 5.67 (minimum = 1.0; maximum = 21). Table I summarizes the characteristics of the study subjects.

**Table I.** Demographic information of study subjects

Variables	
Age	56.92 (SD = 5.82)
Period of attending gyms (years)	15.23 (SD = 8.92)
Hours of physical exercise weekly	5.67 (SD = 5.01)
Gender Male Female	2 (13.3%) 13 (86.7%)
Marital status Single Married	1 (6.7%) 14 (93.3%)
Years of receiving education ≦ 9 years 12 years ≧ 14 years	2 (13.3%) 7 (46.7%) 6 (40%)
Reasons for attending gym Making good us of time Interesting Making self-development Making friends Improving physical health Making good body-shape	4 (26.7%) 7 (46.7%) 3 (20%) 2 (13.3%) 13 (86.7%) 7 (46.7%)
Health problems Cardio-vascular disease Arthritis None	5 (33.3%) 1 (6.7%) 9 *60%)
History of sport injury Sprain Strain Contusion None	2 (13.3%) 8 (53.3%) 1 (6.7%) 4 (26.7%)

### Study findings

**Figure 1.**
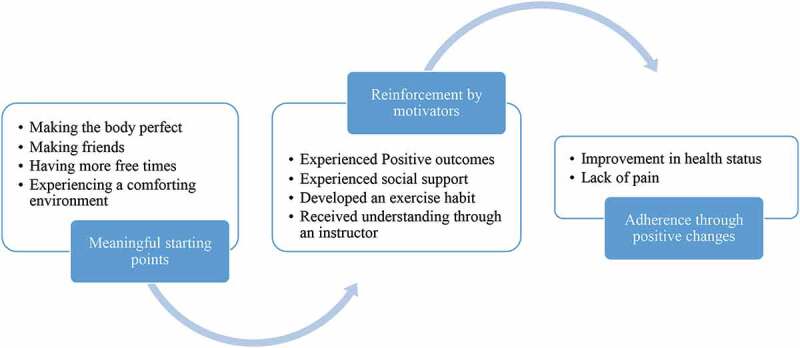
Process of adherence regular physical exercise: Constant daily exercise to keep the doctor away

Figure 1 summarized the process of adherence regular physical exercise, which is the major theme of this study is that “constant daily exercise keeps the doctor away”. Participants perceived that regular physical exercise made them healthy. Subject 2 reported, “I like to attend exercise classes. Although I need to pay fee to attend the exercise classes in the gym, it [outcomes of regularly exercise] is better than to visit a doctor.” Subject 11 also indicated that “no matter it is a sunny day or rainy day, I always attend exercise classes if possible ….I hope to maintain healthy. I don’t want to gain weight because I am a middle-aged person. … I was diagnosed with high blood pressure. I want to control blood pressure [to be normal]. Regular physical exercise can make me feel better [because blood pressure is well-controlled].”

Three categories emerged from the interviews regarding the adherence to regular physical exercise: (1) a meaningful starting point, (2) reinforcement by motivators, and (3) adherence through positive changes. The category of “a meaningful starting point” was defined as what motivated the subject to join a gym and attend exercise classes regularly. This category was divided into four subcategories: (1) making the body perfect, (2) making friends, (3) having more free time, and (4) experiencing a comforting environment. One participant (participant 5) stated that “I am looking for a place where I don’t need to worry about air pollution, or it is a raining day, when I am doing exercise … . A place makes me feel comfortable, then I will go and do exercise regularly … . I can make body sharp perfect if I do exercise regularly. That is why I am here [a gym].” Participant 2 stated that “I retired, I have more free time … . And, a friend of mine asked me to come here [a gym] and to do exercise together … . That is why I am here [in gym].”

The category of “reinforcement by motivators” was defined as including factors or experiences that had an effect on the participants’ continuing to exercise regularly. Four subcategories were defined: (1) experienced positive outcomes, (2) experienced social support, (3) developed an exercise habit, and (4) received understanding through an instructor. One participant (participant 8) indicated that “exercise makes me happy and healthy. When I feel muscle sore, I will do stretch out. That makes me feel more relaxed and happy due to pain release.” Another participant (participant 15) stated that “I improve health due to regular exercise here [gym] … . Exercise makes me happy, heathy and relax. I also make a lot of friends … . My husband also has been very supportive … . Sometimes, he takes care of children, so I am able to attend the aerobics class … . Because my husband and I love to attend exercise classes. Due to our influence, my children love to exercise too … . Doing exercise regularly not only become my habit, but also a habit of my whole family.” Participant 14 indicated that “exercise with friends makes me happy … . I usually like to attend a class, if the instructor can offer multiple class-time options, and if he/she has a well-organized teaching strategy. The instructors in this gym are also charming enough to attract students without absence. That is why I am here and do exercise regularly for many years.”

The category of “adherence through positive changes” was defined as the changes that the participants experienced that led them to attend physical exercise classes regularly. The positive changes included two subcategories: (1) improvement in health status and (2) lack of pain. Participant 3 indicated that “doing exercise regularly improves my body health … . For example, I reduced body soreness. My immunity is improving [less cold and sickness]. I also feel physically comfortable.” Another participant (participant 13) indicated that “doing exercise regularly makes my vitality gradually better. Many outcomes of my physical examination are encouraging and some reach the standard, which enable me to have higher motivation to maintain exercise regularly.”

## Discussion

“Constant daily exercise keeps the doctor away” was the major theme of the current study. The average period of attending a gym were 15.23 years among study participants. The majority of them were female and receiving support from their family members. They adhered to physical exercise to maintain or improve health. These are facilitators to motivate them to adherence to an exercise program. This principal result is consistent with several previous studies. For example, Stodle et al. (2019) conducted a qualitative study of seven older adults to understand the experience of motivation and adherence to group-based exercise classes among Norwegians aged 80 years and older. Their results indicated four main themes: (1) experience of health challenges (a meaningful starting point), (2) adherence motivated by increased life manageability, (3) comprehensibility through skilled instruction, and (4) social and professional support enhancing motivation. Subjects who participated throughout the entire group-based exercise intervention experienced improved health and function as well as to their life situation. The participants have had positive changes in physical, mental, and social functions, and as a result, they have improved their motivation to adhere to regular exercise and positive behavioural change, which are important to their everyday lives.

Similarly, Marcus-Varwijk et al. (2019) conducted a qualitative study to understand the views and experiences of 19 Dutch older adults aged 62 to 92 years who were participating in a nurse-led health promotion intervention, called the Community Health Consultation Offices for Seniors (CHCOS). They found that the main themes among older adults participating in the CHCOS were (1) awareness of ageing, (2) experienced an interaction with the nurse, and (3) perception of the consultations as a check-up and/or personal support. Ageing individuals experience discomforts and physical, mental, and social constraints. The participants reported that they needed to adopt a healthy lifestyle and anticipated new opportunities and constraints in life. These individuals had practised a certain routine for many years, including participating in physical exercise, maintaining social contacts, and refraining from smoking. The individual experiences with and holistic perspectives about healthy living are important factors that influence one’s willingness to engage in and change their health-related behaviours when needed. Moreover, Gothe and Kendall (2016) used three focus groups to investigate the barriers, motivators, and preferences for physical activity among elderly female African Americans. They also found that primary motivators were health and well-being, social support, and enjoyment, which is in agreement with the findings of the present study. Wu et al. (2016) investigated the needs required in gyms and fitness centres for senior citizens in Taiwan. Twenty-six senior citizens participated in four trial exercise programs, including cardiovascular training, resistant training, group exercise, and spa at a gym. The majority of subjects were female (69.2%). Results found that seniors expressed their needs of concerns relating to the centres’ physical environment and professional services during the period of exercise trail. The physical environment of centre included sufficient space, places for rest, adaptable and easy-to-read instructions at training stations, and a stable, non-slippery floor. The professional services included fitness level–based classes and considerate and competent fitness professionals. Thus, despite where the support comes from, previous and present studies supported that receiving support and gender difference might affect an individual to do physical exercise regularly.

Richardson et al. (2017) investigated the gym as a potential place to promote health for individuals with physical disabilities. Twenty-one subjects with physical disabilities were interviewed regarding their experiences with the gym. Their results showed that “experiencing enhanced wellness” was one of the key themes regarding the gym experience. Subjects reported that they were motivated to begin attending a gym and engaging in physical exercise because they believed it would result in improved physical health and enhanced social life and would provide a psychological respite. These findings were similar to those of the present study. However, one of their findings was different from ours, which might be a result of the differences in study populations. Richardson and co-authors found that the gym environment could be a barrier for people with physical disabilities. Subjects noted that they were unable to reach typical physical fitness (such as being strong, muscular, and aesthetically pleasing), which was usually valued in the gym. This caused them to feel incohesive and discomfort towards the gym.

### Limitation of the study

This study has four limitations. First, although study subjects have many years’ experiences of using a gym, they are not expert panellist that we can accept their comments as the scientific truths. Second, subjects used a gym might be related to physical health issues. In this study, the assertion of the participants could have been verified (triangulated) by clinical data like the blood pressure for the participants that confirmed he/she is hypertensive. Third, a non-random recall bias might exist due to opinions of the participants which will be subjective in favour of gym usage. Thus, data of method triangulation may be used in further investigation. Fourth, the participants of the study were Taiwanese individuals. The regional culture might influence individual perceptions of using gym and affect outcomes of the study.

## Conclusion

The gym is a space where individuals can improve their health to reach a status of controlled comfort. It also offers a safe environment and access to qualified instructors. This study showed that the participants reinforced their popular knowledge by their perceptions of “a person’s health condition can be improved via adherence to regular physical exercise.” These findings provide instructors with some guiding principles to design physical exercise classes of higher exercise effectiveness. The principles include providing a comforting environment, providing a supportive instructor via assessing participants’ ability thoroughly, using a well-organized teaching strategy to design a class. These findings can also be used to design and implement further research aimed at increasing adherence to physical exercise among middle-aged and older individuals. To promote health-enhancing behaviours, the results of this study can be applied by health policymakers or managers of gyms/fitness centres to design interventions for community-dwelling people.
